# Measurement of implementation components ten years after a nationwide introduction of empirically supported programs – a pilot study

**DOI:** 10.1186/1748-5908-7-49

**Published:** 2012-05-31

**Authors:** Terje Ogden, Gunnar Bjørnebekk, John Kjøbli, Joshua Patras, Terje Christiansen, Knut Taraldsen, Nina Tollefsen

**Affiliations:** 1 Norwegian Center for Child Behavioral Development, University of Oslo, P.O. Box 7053, Majorstuen, 0306, Oslo, Norway

**Keywords:** Implementation measurement, Implementation components, Empirically supported treatment programs, Parent management training, Multisystemic therapy

## Abstract

**Background:**

Ten years after the nationwide dissemination of two evidence-based treatment programs, the status of the implementation components was evaluated in a cross-sectional study. The aim of the study was to pilot a standardized measure of implementation components by examining the factor structure, the reliabilities of the scores, and their association with implementation outcome variables. The aim was also to compare implementation profiles of the two evidence-based programs based on multi informant assessments.

**Methods:**

The 218 participants in the study were therapists, supervisors, and agency leaders working with Parent Management Training, the Oregon model (PMTO), and Multisystemic Therapy (MST) in Norway. Interviewers filled in an electronic version of the Implementation Components Questionnaire during a telephone interview.

**Results:**

The factor analysis of the eight one-dimensional subscales resulted in an individual clinical-level factor and an organizational system-level factor. Age, experience, and number of colleagues in the workplace were negatively correlated with positive ratings of the implementation process, but the number of colleagues working with the same program predicted positive ratings. MST and PMTO had different implementation profiles and therapists, supervisors, and managers evaluated some of the implementation drivers significantly differently.

**Conclusions:**

The psychometric quality of the questionnaire was supported by measures of internal consistency, factor analyses of the implementation components, and the comparisons of implementation profiles between programs and respondent groups. A moderate, but consistent association in the expected direction was found with the implementation outcome variables.

## Background

Implementation is the movement of evidence-based programs (EBPs) from science to practice, or the active and planned effort to mainstream a new intervention within a practice organization [1]. Implementation also describes a transition period in which practitioners become increasingly skillful, consistent, and committed in their use of a new intervention [2]. Even if new intervention programs are accepted and adopted, they are not necessarily put into practice. This is referred to as the ‘knowing-doing gap,’ in which practitioners fail to do what might improve performance and ‘substitute talk for action’ [3]. Furthermore, when interventions are properly implemented they are threatened by program drift or dilution over time. The rather slow and limited success of transferring EBPs to ordinary service, and the limited impact and sustainability of programs once adoption has occurred, have been the focus of several researchers working with implementation [4-7]. The failure of EBPs to produce expected outcomes may be attributable to the effectiveness of intervention or the quality of the implementation [2]; therefore, both the program effectiveness and its implementation into regular practice should be evaluated.

In a national implementation project initiated in 1999, two treatment programs targeting conduct problems in children and youth were disseminated across all regions of Norway. The Oregon model of Parent Management Training (PMTO) addressed families with children aged 12 years or younger [8], while Multisystemic Therapy (MST) was offered to families with juvenile delinquents in the age range of 13 to 17 years [9]. Randomized controlled replication studies were conducted of both PMTO and MST with encouraging short- and long-term clinical outcomes [10]. A retrospective case study of the Norwegian national implementation of these programs was conducted after eight years [10], but no systematic implementation assessment system was at hand. The increased demand for implementation research has created a need for instruments measuring implementation components across programs and across different stages of implementation. In the present study, the ‘measure of implementation components’ [11] was piloted on the two EBPs that had been implemented in Norway over a period of ten years, making it both a validation study and a study of large scale program sustainability. The implementation components were evaluated by interviewing therapists, supervisors, and agency leaders working with the programs in regular practice.

Implementation research has focused on a string of variables related to the process of transforming research findings into practice, often concluding that ‘everything matters’ [12,13]. The incentives and barriers are often described in terms of attitudes (e.g., openness to change), motivation (e.g., readiness), values (e.g., learning orientation) and other characteristics of adopters, implementers, or stakeholders. Even if research has investigated which organizational structures and mechanisms that mediate or moderate implementation efforts, we still need to know more about the factors that influence organizations’ adoption of programs [14]. Klein and Sorra [2] recommend that rather than searching for critical determinants of implementation effectiveness, researchers should try to document the cumulative influence of all the components. In the same vein, Greenhalgh et al. [1] criticized most studies for focusing on a limited number of implementation components rather than examining them more broadly. Moreover, Fixsen et al*.*[11] were critical of recent implementation studies because the very general measures used did not specifically address core implementation components. According to Fixsen et al*.*, some measures were program specific and lacked generality across programs and some measures only indirectly examined the core implementation components. In contrast, their model focuses on core components at different stages of the implementation process and covers both the individual and organizational level of implementation [15].

### Implementation models

Ideally, conceptual models of implementation should include clearly defined constructs, a measurement model for these constructs, and an analytical model describing the links between the constructs [1,6,15]. In linear stage models, implementation is the final stage in a two-step, one-way linear process: first from basic science to intervention development and testing and second from intervention development to implementation in real world practice settings. In stage models, little is said about the organizational and practice contexts. Alternatively, multi-level models of change differentiate between the large system or policy level, the organization level, the group/team level, and the individual level [16]. The component model of Fixsen et al. [4,15] combines the stage and multi-level model perspectives by describing the temporal progression of the implementation process in six, recursive stages at individual group and organizational levels.

### Implementation stages

Fixsen et al. [15] identified six stages of program implementation, including exploration, installation, initial implementation, full implementation, innovation, and sustainability. The level and quality of implementation should ideally be measured at each of the implementation stages, and the relative importance of each implementation component should be measured and compared across stages in a prospective design. Full implementation is the stage when at least 50% of the positions are filled with practitioners who meet the fidelity criteria. The innovation phase occurs after one or two years of full implementation with acceptable fidelity, and after outcomes of adaptations are carefully evaluated [11]. Sustainability is the final stage in which competence and integrity are maintained in the face of new challenges related to staff turnover and contextual changes. According to Fixsen et al. [17], ten years is approximately the right time to ‘follow up’ on implementation success or failure within an agency; this matches the timeline of the programs examined in the present study.

### Implementation components

Implementation components are hypothesized to help practitioners use innovations in an effective way [15] and are presented in the ‘*Measures Of Implementation Components Of The National Implementation Research Network Frameworks*’ [11]. The components are specific to ‘best practices’ extracted from meta-analyses and primary studies and the authors’ interactions with developers and purveyors of EBPs. The implementation drivers are recruitment, initial training, supervision/coaching, performance assessment, decision support data systems, facilitative administration, systems interventions, and leadership. The last component was added based on recent advances in the study of leadership as an important aspect of implementation [18,19]. The eight implementation components are assumed to be interactive, integrated, and compensatory in such a way that weakness in one component might be overcome by strengths in others. The components and their outcomes exist independently of the quality of the program being implemented, and a good implementation strategy is of little avail without effective interventions [15].

### Implementation strategies of MST and PMTO

All MST therapists have full-time positions and are members of permanent teams. They work exclusively with MST. PMTO, on the other hand, have trained therapists who devote 20% to 100% of their position to the treatment program and may have other responsibilities as well. PMTO is therefore established as a flexible and decentralized program service embedded in local child welfare and child mental health services. PMTO therapists outnumber MST therapists by 383 to 58. Compared to PMTO, MST has a more tightly organized team structure that consists of 23 teams in the specialist child welfare services at the county municipal level. MST was introduced in Norway by a well-organized purveyor organization, the MST Services in Charleston, which supported the implementation by conducting information meetings, site assessments, initial therapist and supervisor training, booster sessions, and monitoring of program and treatment adherence. MST Services had limited experience with implementation of the program outside of the US, and Norway was the first country to implement MST on a national scale [9]. Until 1999, PMTO had almost exclusively been applied for research purposes at the Oregon Social Learning Center. Although it had been developed and evaluated for efficacy, no comprehensive implementation strategy or dissemination organization was established for the program. When invited to implement PMTO nationwide in Norway, the developers in collaboration with the Norwegian implementation team worked out an implementation strategy and model that later has been used in other large scale implementations of the program [8,20]. Compared to MST, PMTO had more therapists and more sites (agencies and organizations) adopting the program, and also greater variations in the time available for each therapist to deliver program services. This greater program context and therapist heterogeneity may account for the greater variations in the assessments of the PMTO implementation see also [21]. In sum, the differences in implementation profiles between the two programs were likely due, in part, to the more uniform implementation strategy of MST.

In the present study, the implementation components were measured retrospectively ten years after the introduction of MST [22] and PMTO [20] in Norway. To the authors’ knowledge, few countries have had such a long-term and extensive experience with nationwide implementation of empirically supported programs. The respondents were therapists, supervisors, and agency leaders working with MST or PMTO.

### Aims of the study

The primary aim of the present study was to examine the factor structure and reliabilities of scores on an adapted and translated version of the Measures of Implementation Components [11]. This questionnaire had not yet been tested in a quantitative study. The study allowed for comparisons between program implementation profiles (MST versus PMTO) and respondents (therapists, supervisors, and agency leaders). The study also investigated the validity of the scores on the drivers through its associations with scores on variables related to implementation outcomes.

## Method

### Participants and procedures

The 218 participants in the present study were recruited from a group of practitioners working with PMTO and MST and consisted of: trained and experienced PMTO and MST therapists (n = 149); supervisors (n = 45) of the therapists who participated in the study; and agency leaders (n = 24) who were making decisions and were responsible for the overall organization or the part of the organization in which the therapists worked. A computer-generated random sample of 100 therapists was selected among the certified PMTO therapists who were active in the child welfare or child mental health specialist services. Among these, 93 participated together with all registered PMTO supervisors (n = 24). Among the PMTO therapists, a random selection of 20 of their leaders were chosen for interview and, of those, 13 participated. Fifty-six of 58 MST therapists, and all MST supervisors (n = 21) and leaders (n = 11) contributed to the study.

Among the therapists, 109 out of 149 participants were women (73.2%), among whom 93 worked with PMTO and the remaining 56 with MST. Their age range went from 26 to 64 years with a mean age of 46 years. When the study was carried out, some of the respondents had just started in their jobs and some had been in their position for 32 years, with a mean of 4.5 years in their present job. All therapists were trained between 1999 and 2009, and the median period of working with the programs was three years. The therapists reported that their median number of colleagues were 15. A median number of three worked with the same program as the therapists, and the median number of colleagues working with another EBP was one. The MST therapists had between 2 and 10 colleagues working with the same program (median = 4, which corresponds with the size of most MST teams) and 21 PMTO therapists had no colleagues working with the same program. Approximately 50% of the therapists, mostly PMTO therapists, had no colleagues working with EBPs.

### Questionnaires and interviews

The data collection took place during December 2009 and January 2010 and was organized as a procedure where the interviewers filled in an electronic questionnaire on the internet during a telephone interview. The interviews were carried out by ten MST and twenty PMTO program experts affiliated with the Norwegian Center for Child Behavioral Development. Requests for participation were e-mailed to the respondents, and upon confirmation, appointments were scheduled and the respondents received the questionnaire in MSWord format prior to the interviews. Each interview lasted for approximately sixty minutes.

### Measure

The Implementation Components Questionnaire (ICQ) was adapted from the Measures of Implementation Components of the National Implementation Research Network Frameworks [11]. Adaptation of the measure involved translation from English to Norwegian, and rewording of certain questions to apply to the sample. Each item had three response alternatives in which ‘No’ = 0, and the two remaining response alternatives (‘yes’ and ‘sometimes’) were collapsed into one, and scored as 1. It was assumed that by using the ‘yes’ and ‘no’ format, the answers would be more reliable and reflect whether the actual indicator was present or not. The two additional categories, ‘not relevant,’ and ‘I don’t know.’ were scored as missing. A brief description of each of the eight scales is provided in the results section of this article.

The implementation climate scale consists of 32 items that relate to the use of innovations in organizations and were adapted from the work of Klein and Sorra [2] and Panzano et al*.*[18]. In line with the rest of the ICQ questionnaire each item had three response alternatives in which ‘No’ = 0, and the two remaining response alternatives (‘yes’ and ‘sometimes’) were collapsed into one, and scored as 1.

For the implementation outcomes questions, the therapist respondents were asked to rate the perceived level of integration of the program within their organization, their overall satisfaction with the implementation process, how much time they spent working with the program, their productivity in terms of the number of cases they had treated with the program, and the number of families who completed treatment. Finally, they responded to the statement ‘sooner or later I am going to quit using the program.’

### Analyses

The implementation items were measured at an ordinal scaling level and analyzed by Categorical Principal Component analysis (CATPCA) using the PASW Statistics 18 software (2010; formerly SPSS). ‘While Principal Component Analysis (PCA) assumes that the variables used are metric, and proceeds to the spectral decomposition of the correlation matrix, CATPCA relate on an alternative least squares scheme iterating between quantification and a decomposition phase’ [23]. A Cronbach’s alpha is calculated for each retained dimension [24]. Due to the small sample size in relation to the number of variables, CATPCA was only conducted on the items for each driver separately. For each driver, we first explored a two dimensional solution to test if there was a clear discernible pattern of items loading on the second dimension before we explored the expected one-dimensional solutions. To retain an item in a factor, two criteria were applied: the absolute value of the weighting was equal to or higher than 0.40; and all quantified ordinal variables correlated 0.50 with at least one of the components. Following a rule of thumb for standard PCA, fulfilling both criteria meant that all items contributed well to the description of the characteristics of our sample and all items were sufficiently correlated to one another to be useful in the analysis [25], p.128. If either criteria were not fulfilled, the item was excluded. Because of the rather high rates of respondents included with one or more missing values on the scored variables, it was decided to use CATPCA option of treating missing values as an extra category [26]. This option implies that the missing category will obtain a quantification that is independent of the analysis level of the variable. The greatest advantage of this in our study was that it enabled us to deal with variables that include categories like no response, don’t know, or not applicable. Mean scale scores were computed for the eight implementation components after the items were trimmed. The scales scores were then examined using descriptive analyses and bivariate correlations. Finally, differences between informants (therapists, supervisors, and agency leaders) and programs (MST and PMTO) were investigated.

## Results

The CATPCA analysis showed that for all implementation component scales, the Eigenvalues dropped substantially from the first to the second dimension suggesting only one-dimensional solutions. As an indicator of internal consistency of the dimensions, Cronbach’s alphas were analyzed. For the expected one-dimensional solutions, the alphas ranged between 0.79 and 0.91. In the two-dimensional solutions, the alpha was considerably lower, ranging from 0.09 to 0.65, and was higher than 0.35 only on the supervision driver. Moreover, on all first components, items loaded positive, whereas this was not the case on the second components. The loadings on the second component were rarely larger than those on the first, not pointing to a unique contribution to the second dimension. Thus, Cronbach’s alpha, variance explained, and scree tests based on eigenvalues indicates one-dimensional solutions.

### Recruitment - practitioner selection (nine items)

The items in this scale focus on how staff is recruited to work with the program during its initial phases of implementation, and how staff is recruited to help sustain the program over time. One factor was extracted after using a CATPCA analysis. All items loaded satisfactorily on the recruitment factor (from 0.41 to 0.90). The factor accounted for 53% of the total variance. Reliability for the scale was 0.89. The items in this scale appeared to be the most difficult to answer based on rather high rates of missingness (84 missing, 39%), which was particularly true for therapists.

### Training (ten items)

This scale focuses on activities related to providing information, instruction, or skill development to practitioners and other key staff in the implementing organization. This is distinct from the supervision/coaching scale in that the training scale focuses on the initial acquisition of key skills related to the program. One item from the original scale was excluded due to lack of adequate loadings (i.e., <0.40). A rerun of the component analysis using only the selected ten items produced a one-dimensional solution with loadings ranging from 0.41 to 0.97. The training factor accounted for 56% of the total variance. Reliability for the scale was 0.91. The distributions of some of the items from the scale were highly non-normal (see Table 1).

**Table 1:** Descriptive statistics for implementation drivers

						Skewness		Kurtosis	
	*n*	Min.	Max.	*M*	*SD*	Statistic	*SE*	Statistic	*SE*
Recruitment	143	.00	1.00	.5874	.27522	-.687	.203	-.445	.403
Training	165	.40	1.00	.7545	.14418	-.154	.189	-.559	.376
Supervision	215	.00	1.00	.7881	.19485	-.631	.166	.089	.330
Perform. assess.	184	.00	1.00	.6103	.28217	-.550	.179	-.756	.356
Datasystem	186	.00	1.00	.5848	.28464	-.426	.178	-.552	.355
Administration	171	.00	1.00	.4277	.33431	.124	.186	−1.322	.369
System interv.	206	.00	1.00	.7852	.19533	−1.244	.169	1.628	.337
Leadership	204	.00	1.00	.6020	.25619	-.520	.170	-.468	.339
Total	210	.12	1.00	.5636	.20170	-.049	.168	-.750	.334
Valid N (listwise)	83								

### Supervision/coaching (nine items)

The questions on this scale focus on the ways that supervisors provide guidance to therapists and practitioners and also how often, where, and how feedback information is collected. There was little variation in the answers on these items. Two items from the original scale were excluded due to lack of adequate loadings. A rerun of the CATPCA analysis using only the selected nine items produced a one-dimensional solution with loadings ranging from 0.52 to 0.67. The factor accounted for 37% of the total variance. Reliability for the scale was 0.79.

### Performance assessment (ten items)

This scale is related to the type, frequency, and method of performance evaluation of the practitioners in relation to their use of the program. Specifically, questions deal with measures of integrity to the method, as well as how often and by whom their performance is evaluated. All items loaded satisfactory on the performance assessment factor (from 0.48 to 0.84) and the factor accounted for 50% of the total variance. Reliability for the scale was 0.89.

### Decision support data systems (nine items)

This scale is based on the availability of information through systematic acquisition of data. The goal of these systems is to provide feedback to stakeholders, therapists, coaches, and policy makers inside and outside of the organization. All items loaded satisfactory on the factor (from 0.47 to 0.75) and the factor accounted for 44% of the total variance. Reliability for the scale was 0.84.

### Facilitative administration (seven items)

These items tap into whether those in charge of implementation in the host organization had restructured and adapted the organization to make implementation and sustainability successful. All items loaded satisfactory on the facilitative administration factor (from 0.51 to 0.82) and the factor accounted for 48% of the total variance. Reliability for the scale was 0.82. It is likely that many of the questions were difficult for the therapists to answer because data was missing for 39 to 78 of the 149 respondents.

### Systems interventions (twelve items)

The items in this scale are based on the participants’ organizations work to influence the systems and policies in their region to develop better support for the innovation. All items loaded satisfactory on the system intervention factor (from 0.41 to 0.74) except for item seven. The twelve-item factor accounted for 36% of the total variance. Reliability for the scale was 0.82.

### Leadership (fifteen items)

The questions about leadership focused on ways different people within the organization engaged in leadership behavior and provided systematic support, clear communication with practitioners, provided decision-making, garnered feedback, and engaged actively in a manner that was conducive to successful program implementation. All items loaded satisfactory on the leadership factor (from 0.41 to 0.74) and the factor accounted for 37% of the total variance. Reliability for the scale was 0.88.

### Therapist assessment of implementation components

The following is a presentation of the associations between therapist background characteristics and implementation assessments and a more detailed description of therapist ratings of the eight subscales. Although the programs and their implementation strategies and structure differ, they give a snapshot of the sustainability of the implementation components in two EBPs that have been in operation for approximately ten years.

### Background characteristics

The associations between the therapist implementation ratings and their background characteristics were calculated and the mean total implementation, that is, the mean of all implementation components, were negatively correlated with age (r = −0.21, *p* < 0.01), with the number of years working (r = −0.20, *p* < 0.05) and the number of colleagues (r = −0.37, *p* < 0.001) in the present position, and positively correlated with the number of colleagues working with the same program (r = 0.44, *p* < 0.001). In other words, the older the therapists were, the more time they had worked as therapists, and the more colleagues they had, the more they tended to give low ratings of the implementation components. The openness to change may vary among practitioners, and the older and more experienced among them may have been less motivated to incorporate an evidence-based method in their daily work. Based on their longer field experience, some of the older practitioners may also have had higher expectations for the implementation process. Given the rather low level of general support for the evidence-based movement in the practice field, the organizational climate in large agencies may have been less supportive of the new program. The respondents with relatively many colleagues may have faced more difficulties when they introduced the principles and practice of an empirically supported program. Both their age and experience may have contributed to higher expectations for implementation support, particularly at the organizational level.

On the other hand, the more program colleagues the therapists had, the more positive ratings they tended to give. However, the number of years working with the program was not predictive of the implementation evaluation.

### Descriptives

The mean scores for the scales were from 0 to 1. Descriptives of all scales revealed that the mean scores were at the upper end of the scale, with the exception of administrative support (M = 0.43, SD = 0.33) (see Table 1). The mean total implementation score was approaching normality with a mean of 0.56 and standard deviation of 0.20. The values of skewness and kurtosis were within acceptable limits for all subscales and items and suggested that the data were univariate non-normal (see Table 1) [27,28].

### Correlations

Bivariate Pearson’s correlations between the eight component scales were computed to examine the relationship between the scales and are presented in Table 2. The correlations between the training scale and the other drivers were in the small to moderate range (ranged from 0.12 to 0.30). The relationship between leadership and facilitative administration, system interventions, and decision support data systems scales were of medium size (ranged from 0.31 to 0.40). The associations between leadership and recruitment, supervision and performance assessment were non-significant. The systems interventions scale showed moderate associations with the other drivers (ranged from 0.30 to 0.38), with exception of a non-significant correlation between systems intervention and training and recruitment. The association between recruitment and supervision and performance assessment, between performance assessement and supervision, and between facilitative administration and decision support data systems were large and ranged from 0.50 to 0.70 (small correlation = 0.10, medium = 0.30, large correlation = 0.50; 32).

**Table 2:** Pearson’s bivariate correlations between seven of the eight implementation drivers

	1.	2.	3.	4.	5.	6.	7.	8.
1. Recruitment	1							
2. Training	.27**	1						
3. Supervision	.70**	.17	1					
4. Performance assessment	.58**	.26**	.66**	1				
5. Decision support data	.30**	.28**	.33**	.41**	1			
6. Facilitative administration	.50**	.30**	.47**	.42**	.50**	1		
7. Systems interventions	.14	.12	.35**	.30**	.40**	.38**	1	
8. Leadership	-.14	.23**	-.09	.03	.31**	.38**	.40**	1

Note. **. Correlation is significant at the 0.01 level. *. Correlation is significant at the 0.05 level.

The pattern of the correlations between the implementation drivers indicated that the drivers could be divided into an individual-clinical level and an organizational-system level. Therefore, a principal component analysis (PCA) was conducted on the implementation components and oblimin rotations were examined. The analysis yielded two factors with eigenvalue exceeding unity (see Table 3). The first factor accounted for 48% of the variance and consisted of the organizational-system level subscales of leadership, administrative support, decision support data systems, and system intervention. The second factor accounted for 17% of the variance and consisted of individual-clinical level subscales of supervision, training, recruitment, and performance assessment. Thus, the results from this study indicate a two-factor structure representing implementation components at the individual clinical level that the national center was mainly responsible for and the organizational system level that to a large extent was under the control of the local adopting organizations. The ‘decision support data system,’ ‘recruitment,’ and the ‘supervision’ subscales had a high pattern coefficient on both factors. However, all variables loaded above 0.50 on their primary factor.

**Table 3:** Structure and Pattern Coefficients (in Parentheses) of the Variables in the Oblique-rotated 2-factor Solution

Scale:	Organizational system level	Individual, clinical level
Facilitative administration	**.97 (1.00)**	.23 (−.03)
Decision support data systems	**.64 (.54)**	.45 (.26)
Systems intervention	**.42 (.41)**	.17
Leadership	**.41 (.44)**	
Performance assessment	.31	**.93 (.93)**
Recruitment/selection	.62 (.42)	**.71 (.56)**
Supervision/coaching	.59 (.45)	**.58 (.43)**
Training	.11	**.51 (.54)**

Note. Factor loadings > .40 on their primary component are in boldface.

Prior to running correlations between the outcome variables and organization level and individual level scale variables, only therapist data was selected because the outcome questions did not correspond well to supervisor or agency leaders’ experience. An ‘implementation sum score’ was also calculated by summarizing scores on the eight subscales. Correlations between the outcome variables and scale variables are reported in Table 4. Therapist assessment of the extent to which the program was well integrated into the organization correlated significantly with seven of eight components and with the total score, although in the small to moderate range. The therapists reported how much (in percent) of their position was used on program activities. There was a positive correlation indicating that the larger the proportion of the position therapists worked with the EBP, the more positive the ratings were on the implementation subscales, particularly recruitment, supervision, performance assessment, and the total score (r = 0.57, *p* < 0.01). Another outcome variable of interest was the number of families who completed treatment within the last six months. The findings revealed that scores on recruitment, supervision, and performance assessment were moderately positively associated with the number of families who completed treatment. The recruitment and supervision components were related to how satisfied the therapists were with the implementation progress. Finally, only training and leadership were associated with whether it is likely that the therapist will quit practicing the EBP.

**Table 4:** Pearson’s correlations between implementation drivers and dependent variables

	1.	2.	3.	4.	5.	6.	7.	8.	9.
EBP is well integrated into the organization	.38^**^	.07	.42^**^	.24^**^	.27^**^	.28^**^	.32^**^	.19^*^	.43**
Satisfied with implementation progress	.25^**^	-.03	.26**	.12	.05	.17	.11	.04	.19*
Sufficient number of cases	.07	.08	.16*	-.01	.05	-.15	.09	.04	.05
Percent of position to work with EBP	.76^**^	-.02	.64**	.50^**^	.21^*^	.37^**^	.23^**^	-.01	.55**
Likely to stop using EBP	.01	-.23^*^	.11	.05	-.14	-.14	-.07	-.23^**^	-.04
No. of families referred last 6 mos.	.16	.00	.18^*^	.12	.14	-.05	.09	-.01	.16
No. of families who completed treatment – last 6 mos.	.40^**^	.06	.30^**^	.26^**^	.14	.11	.07	-.05	.30**

1. Recruitment and staff selection, 2. Training, 3. Supervision, 4. Performance assessment, 5. Decision support data systems,

6. Administrative support, 7. Systems interventions, 8. Leadership, 9. Implementation sumscore.

Note. **. Correlation is significant at the 0.01 level (2-tailed), *. Correlation is significant at the 0.05 level (2-tailed).

As noted, we created two scales based on the eight components, namely an organizational system factor and an individual, clinical factor. Table 5 shows that the two subscales were significantly correlated (from small to moderate) with most outcome variables. Exceptions were that the individual subscale had a large correlation with the proportion of the therapists’ position devoted to work on the EBP and the organizational subscale had a large correlation with implementation climate. The organizational factor was not significantly correlated with how many colleagues the interventionist had in the present position and the individual factor was not correlated with whether it is likely that the therapist will quit practicing the EBP.

**Table 5:** Correlations – implementation factors and dependent variables

**Dependent variables:**	**Organizational system factor**	**Individual, clinical factor**
Overall satisfaction with the implementation process	.15*	.24**
How large part of position set aside to work with families	.19**	.58**
EBP is well integrated into the organization	.28**	.34**
Sooner or later I am going to quit practicing MST/PMTO	-.19**	.07
Colleagues working with the same program	.22**	.49**
Colleagues in the present position	-.08	-.39**
No. of families who completed treatment – last 6 mos.	.15*	.35**
Implementation climate	.65**	.23**

Note. ** Correlation is significant at the 0.01 level (2-tailed), * Correlation is significant at the .05 level.

### Comparing informants

ANOVAs and *post hoc* tests (Bonferroni) showed that there were significant differences between the three respondent groups and mostly between therapists and supervisors. Significant differences between therapists and supervisors were demonstrated on the following subscales: Recruitment and staff selection, F(2,142) = 7.10, *p* = 0.001, training, F(2,164) = 5.99, *p* = 0.003, supervision, F(2, 214) = 6.14, *p* = 0.003, reporting of results, F(2, 183) = 7.13, *p* = 0.001, administrative support, F(2, 170) = 8.03, *p* = 0.000, and system interventions, F(2, 205) = 11.05, *p* = 0.000. On two of the subscales, the therapists scored significantly lower than both the supervisors and the leaders. The first was decision support data systems, F(2, 185) = 20.88, *p* = 0.000 in which the therapists had a mean score of 0.50 (SD = 0.27) the supervisors had a mean score of 0.77 (SD = 0.26) and the leaders had a mean score of 0.72 (SD = 0.16).Therapists also scored leadership lower than leaders, F(2, 203) = 9.79, *p* = 0.000; therapists had a mean score of 0. 55 (SD = 0.25), the supervisors had a mean score of 0.69 (SD = 0.25), and the leaders had a mean score of 0.75 (SD = 0.19).

### Comparing PMTO and MST

The mean scores for the total scale proved to be significantly different between the PMTO and MST groups, and significant differences in favor of MST were registered for therapists, supervisors, and leaders. The scores reported by PMTO therapists (see Table 6) were lower for recruitment, supervision, reporting of results, decision support data systems, administrative support, and system interventions than scores reported by MST therapists; however, PMTO therapists’ scores were higher than MST therapists on the leadership component. The score on individual system factors were significantly higher for MST therapists than for PMTO therapists, however, the difference in organizational system scores was not significant.

**Table 6:** Mean, standard deviations and *t*-test of differences between PMTO and MST

	**PMTO**		**MST**				
	Mean	SD	Mean	SD	t-value	Sig	N
Recruitment	.34	.26	.69	.13	−8.30	.00	92
Training	.72	.14	.74	.13	-.77	.44	108
Supervision	.64	.15	.97	.06	−15.09	.00	148
Perf. Assessment	.37	.23	.78	.14	−11.53	.00	121
Data systems	.44	.28	.58	.23	−2.79	.00	121
Administrative supp.	.30	.30	.55	.29	−3.77	.00	111
Systems interventions	.70	.21	.81	.17	−3.09	.00	140
Leadership	.60	.23	.46	.26	3.20	.00	148
Organizational system	.55	.22	.59	.21	−1.81	.07	208
Individual, clinical	.44	.20	.80	.12	−15.19	.00	205

## Discussion

The aim of this study was twofold: first,to pilot the Implementation Components Questionnaire (ICQ) which was adapted and translated from Fixsen et al*.*[11] by examining the factor structure, the reliabilities of scores, and their association with implementation outcome variables. The second aim was to analyze and compare implementation profiles of two EBPs based on assessments by therapists, supervisors, and managers.

The ICQ was tested in a sample of 218 MST and PMTO professionals (therapists, supervisors, and agency leaders) in Norway in the ‘sustainability’ phase of implementation, ten years after their initial implementation. The psychometric qualities of the questionnaire were supported by measures of internal consistency, the factor analyses of the implementation components, and the comparisons of implementation profiles between programs and respondents. There was also a moderate but consistent association between component scores and implementation outcomes in the expected direction.

Overall, the respondents reported mean scores at the upper end of the scale for ‘recruitment,’ ‘training,’ and ‘supervision,’ and also for ‘systems interventions’ and ‘leadership.’ Lower mean ratings were given for ‘performance assessment,’ ‘decision support data systems,’ and ‘facilitative administration.’ The total implementation score was also in the middle range, reflecting a normal distribution of ratings when they were pooled across respondents and programs. As reflected in the high mean scores and low variation on the training and supervision scales, both programs seemed to have established and sustained highly structured and consistent procedures for training and supervision. The respondents were generally satisfied with the strategies initiated to influence external systems to contribute with support and resources needed for the local running of the programs. Systems interventions included strategies for ensuring funding and support, routines for referral of cases, and information to families and other local stakeholders. The implementation components of ‘performance assessment,’ ‘decision support data systems,’ and ‘facilitative administration’ reflect new demands faced by the host organizations when adopting EBPs. These dimensions were given the lowest ratings among the subscales, but higher among MST than among PMTO informants, probably reflecting a higher awareness and a more developed strategy for implementing these components in MST. Leadership was the only component that was rated more positively in PMTO than in MST, and may indicate that the decentralized implementation strategy of PMTO may have resulted in a closer relationship between local leaders and therapists in PMTO than in MST.

The factor analysis of seven of the eight subscales demonstrated a two-level factor structure of the implementation components, which is in line with the multi-level perspective discussed in the literature [6,15,16]. The organizational factor included ‘leadership,’ ‘decision support data systems,’ ‘administration,’ and ‘systems interventions,’ which to a large degree are under the control of the program’s host agencies. The individual-clinical factor measured ‘recruitment,” supervision,’ and ‘performance assessment,’ which to a greater extent reflects the contributions from the Norwegian Center for Child Behavioral Development. As a purveyor of the programs, the national implementation teams perform site assessments, give guidelines and advice concerning recruitment of therapists, and continuously conduct training and supervision. The implementation components identified in the current study have many similarities with the concept of ‘technical support’ in Mihalic and Irwin’s [29] evaluation of the Blueprint programs. Both evaluations emphasize the quality of training, supervision, material and handbooks, a system of performance assessment (quality assurance), and good working relations between purveyors and practitioners [1,15].

Among the therapists, the most positive ratings came from young therapists with shorter professional careers who worked more than 80% on the program and in the company of program colleagues. This included tightly organized MST teams, but also team-based PMTO therapists. Ratings were not influenced by the number of years of experience with the program, a finding that attests to the ability of the programs to sustain therapist engagement. Also, the median number of three years of program experience indicated a considerable stability among program staff.

The differences in response patterns among the informant groups may reflect their different experiences and perspectives on the implementation process. The ratings of ‘leadership’ may to a certain extent reflect the respondents positions in the agency hierarchy to the effect that a higher position may have contributed to a more positive evaluation of the quality of the leadership. When the therapists rated several of the implementation components lower than the supervisors, this may be explained by the fact that the supervisors had more organization and system level information than the therapists, and also were more involved in the implementation process. It may also be that the agency leaders had limited experience with the programs, and therefore had to rely on second hand information about the implementation process.

The analyses of outcome variables showed that there was a moderate association between the implementation components and the therapists’ ratings of how well the program was integrated into their local organization. There were also associations between scores on the implementation subscales and the percent of the position set aside to work with the program and the number of families who completed treatment within the last six months. Because the therapist both reported the dependent and independent variables at the same time, the direction of the association is unclear.

### Limitations and future directions

The findings in this study should be interpreted in the context of certain limitations. First, with a few exceptions, the ICQ proved to have acceptable psychometric qualities in the present study. The low internal consistency of the training scale reflected the restricted sample included in the present study, and the ceiling effect found may be explained by the standardized training programs supplied by the purveyor organization. For the same reason, the generalizability of the findings may be limited. The recruitment and training scales likely had the largest number of missing answers because in addition to personal experiences of being employed and trained, they required that the respondents should have a good overview of the program routines for recruitment and training. This may be an indication of the need within each implementation dimension to have some general and some respondent-specific questions. Second, in order to further demonstrate the predictive validity of the ICQ measure, it is necessary to include outcomes (other than those included in this study) that are presumed to be predicted by the implementation components. Implementation outcomes may include the penetration of a program within an organization [6], the increase of productivity and competence, or changes in organizational structures or procedures within an organization. More formal examination of the relationship between the implementation components and performance assessments of PMTO could be examined by including the Fidelity of Implementation Rating System (FIMP), which is an observational fidelity measure specifically developed for PMTO that has been found to predict positive change in parenting practices [30]. Similarly, the relationship between the ICQ measure and Treatment Adherence Measure Scores (TAM scores) [31] should also be examined.

In this study, all informants (therapists, supervisors, and leaders) were affiliated with either MST or PMTO, and this may have been reflected in positive attitudes towards either of the programs and their implementation status. Therefore, and in order to reduce this potential bias, future investigations should include non-affiliated informants or observation data. Future studies should test the ICQ measure in different samples representing other empirically supported programs. Because both PMTO and MST in Norway employ (albeit to different degrees) structured implementation strategies, the findings from this study do not generalize to interventions without clear implementation strategies. Furthermore, the low variance obtained on some of the scales is also probably related to the program’s implementation strategies, although it may be related to instrument sensitivity. This leads to another challenge: Low variance (every participant has almost the same score) does not leave much room for prediction of outcomes. Replication of this study in different samples (e.g., in school-wide programs) is therefore vital in order to further test the validity of the ICQ measure.

The questionnaire introduced a limitation to a reporting period of six months prior to the interview, and this may have introduced confusion, because many of those interviewed had been recruited several years ago. Some interviewers allowed respondents to forego the six-month window, and some probably ignored the timeframe without any explicit permission. This fact made the interpretation of several items difficult. Although the development of the ICQ measure is still in an early phase, it is encouraging that results point in the expected directions: The highest scores were obtained on the scales where the Behavior Center has been most able to influence outcomes, and MST professionals reported higher scores than PMTO professionals on scales where the MST organization has had a clearer and more structured implementation strategy. These findings may reflect the validity of the ICQ measure.

## Conclusion

Rather than being a one-time event, the implementation of EBPs is a process that might take three to four years [15], but may also languish for 15 to 20 years before they are integrated into routine practice [6]. The present study indicates that the strong focus on implementation in MST and PMTO has paid off 10 years after the programs were introduced in Norway by revealing a strong and ongoing presence within agencies, and a relatively long median lifespan of program practitioners. Several incentives for program sustainability, therapist engagement, and treatment fidelity were acknowledged by the respondents in their evaluations of the implementation components.

## Competing interests

The authors declare that they have no competing interests.

## Authors’ contributions

To designed the study, took part in the translation and adaptation of the questionnaire, organized the data collection and conducted the statistical analyses in collaboration with the other co-authors (GB, JK, and JP), interpreted the results, wrote parts of the manuscript and coordinated the efforts of the co-authors. GB conducted several of the statistical analyses, interpreted the results, wrote parts of the manuscript and designed several of the tables. JK conducted several of the statistical analyses, interpreted the results, wrote parts of the manuscript and designed several of the tables. JP conducted several of the statistical analyses, interpreted the results, wrote parts of the manuscript and designed several of the tables. TC assisted in designing the study, took part in the data collection and interpretation of the analyses, and helped to draft the manuscript. KT assisted in designing the study, translating and adapting the questionnaire, took part in the data collection, interpreting the statistical analyses and helped to draft the manuscript; NT assisted in designing the study, translating and adapting the questionnaire, collecting data, interpreting the statistical analyses and helped to draft the manuscript. All authors have read and approved the final manuscript.
